# Phosphine Toxicity: Ethical Questions

**DOI:** 10.1289/ehp.114-a84a

**Published:** 2006-02

**Authors:** Elizabeth L. Anderson

**Affiliations:** *Risk Analysis: An International Journal*, Alexandria, Virginia, E-mail: elanderson@sciences.com

In their article McDaniel et al. (2005) presented three case studies, one involving an evaluation by Sciences International, Inc. (Pepelko et al. 2004), of which I am the president and CEO. This case study was related to the reregistration of phosphine by the U.S. Environmental Protection Agency (EPA). McDaniel et al. (2005) make two principal accusations: *a*) that I improperly used my status as editor-in-chief of *Risk Analysis: An International Journal* (*Risk Analysis*) in the publication of an article on phosphine toxicity, and *b*) that work done by Sciences International led the U.S. EPA to make an improper decision about phosphine risk. There are serious misrepresentations and omissions in this article. Also, neither the authors nor *EHP* contacted me before posting of the article.

Sciences International was engaged in 1998 by a coalition of companies with an interest in the fumigation uses of phosphine to provide an evaluation of phosphine acute toxicity for consideration in the U.S. EPA phosphine reregistration process. The membership of the coalition was diverse, representing industries in food processing, grain milling, rail transportation, and tobacco.

Based on this work, an article was published by scientists at my firm in 2004 on the toxicity of phosphine in *Risk Analysis* (Pepelko et al. 2004). The article was published 5 years after the U.S. EPA made their decision (U.S. EPA 2001) and presented a somewhat more conservative conclusion than that presented by the U.S. EPA. Quite contrary to the impression given by McDaniel et al. (2005), this article went through a thorough peer review and was handled properly in all respects. As a matter of policy, when I, or any member of the editorial staff for *Risk Analysis,* have a potential conflict of interest, we recuse ourselves from the review. Therefore, to avoid any conflict of interest, I asked Curtis Travis, the editor-in-chief emeritus of *Risk Analysis,* to handle the review of the two articles that were submitted in 2002, after the reregistration decision for phosphine. Travis sent the draft articles to independent reviewers and ultimately rejected both articles. His comments included the recommendation to consolidate them into one article. We submitted a revised and consolidated article in 2003, again handled by Travis; the article was accepted and published in October 2004 (Pepelko et al. 2004). McDaniel et al. (2005) made an issue of a suggestion I made that the article (Pepelko et al. 2004) could be expedited in the publication process. It is not uncommon for journals to expedite articles that are of timely interest, such as being relevant to a current decision and particularly in cases of new scientific developments. However, the phosphine article (Pepelko et al. 2004) was ultimately never expedited, a fact that McDaniel et al. did not mention after making the initial accusation. Our article (Pepelko et al. 2004) was handled properly and professionally in all respects.

Secondly, McDaniel et al. (2005) implied that the U.S. EPA improperly selected its uncertainty factors for the phosphine risk assessment based on the analysis done by Sciences International. McDaniel et al. did little to make the case that the U.S. EPA’s decision was improper, other than to point out that not everyone agreed about it. It is notable that our article (Pepelko et al. 2004) recommended an exposure standard of 0.1 ppm, which is lower (more stringent) than the U.S. EPA’s earlier decision (U.S. EPA 2001), and also lower than the standards set by the American Conference of Governmental Industrial Hygienists (ACGIH 2000), the Occupational Safety and Health Administration (OSHA 1999), and the National Institute for Occupational Safety and Health (NIOSH 1997). As described above, our article went through a rigorous peer review and represents a significant scientific contribution; slight scientific differences are not unusual, given the uncertainties involved in setting acute toxicity standards.

McDaniel et al. (2005) provided little description of the ultimate regulatory decision made by the U.S. EPA in regard to phosphine (U.S. EPA 2001), which is necessary to provide context to this discussion. The changes made to phosphine usage were significant, including the requirement for site-specific fumigant management plans, training and certification requirements, and additional label modifications to reduce harmful exposures. These changes represent a significant change in how phosphine is used, substantial requirements and burdens for users, and significant public health protections.

There are legitimate scientific issues that require resolution for setting safe acute toxicity levels, for example, for substances of interest for homeland security. Differing durations of exposure and the accompanying severity of effects present a challenge for evaluating health effects associated with short-term, acute exposures. Investigative tools, including the use of categorical regression and the regional gas-dose model for extrapolating from rat inhalation studies to humans, have been explored by the U.S. EPA and Sciences International for their utility in defining safe acute toxicity levels (U.S. EPA 1994, 2000); the applications of these approaches have been investigated for their utility in setting acute toxicity standards for phosphine. McDaniel et al. (2005) did not attempt to address these challenging scientific issues.

McDaniel et al. (2005) made no attempt to further scientific knowledge; therefore, their article appears to fall short of the scientific standards of *EHP*.
